# Association between ultra-processed foods intake with lipid profile: a cross-sectional study

**DOI:** 10.1038/s41598-023-34451-x

**Published:** 2023-05-04

**Authors:** Mehran Nouri, Sevda Eskandarzadeh, Maede Makhtoomi, Milad Rajabzadeh-Dehkordi, Niloofar Omidbeigi, Maryam Najafi, Shiva Faghih

**Affiliations:** 1 Department of Community Nutrition, School of Nutrition and Food Science, Shiraz University of Medical Sciences, Shiraz, Iran; 2 Students’ Research Committee, Shiraz University of Medical Sciences, Shiraz, Iran; 3 Health Policy Research Center, Institute of Health, Shiraz University of Medical Sciences, Shiraz, Iran; 4 Department of Nutrition, School of Public Health, Iran University of Medical Sciences, Tehran, Iran; 5 Department of Clinical Nutrition, School of Nutrition and Food Science, Food Security Research Center, Isfahan University of Medical Sciences, Isfahan, Iran

**Keywords:** Cardiology, Diseases, Health care, Medical research, Risk factors

## Abstract

The purpose of this cross-sectional study was to examine the association between ultra-processed foods (UPFs) intake and lipid profile in Iranian people. The study was performed on 236 individuals with the age range of 20–50 years in Shiraz, Iran. Food intakes of the participants were evaluated using a 168-item food frequency questionnaire (FFQ) which was previously validated in Iranian populations. In order to estimate the ultra-processed foods intake, classification of NOVA food group was used. Serum lipids including total cholesterol (TC), triglyceride (TG), high density lipoprotein cholesterol (HDL-C) and low density lipoprotein cholesterol (LDL-C) were measured. The results showed that mean of age and body mass index (BMI) of the participants were 45.98 years and 28.28 kg/m^2^, respectively. Logistic regression was used to evaluation the relation between UPFs intake and lipid profile. Higher UPFs intake was associated with increased OR of TG and HDL abnormality in both crude (OR 3.41; 95% CI 1.58, 7.34; P-trend = 0.001 and OR 2.99; 95% CI 1.31, 6.82; P-trend = 0.010) and adjusted models (OR 3.69; 95% CI 1.67, 8.16; P-trend = 0.001 and OR 3.38 95% CI 1.42, 8.07; P-trend = 0.009). But, there were no association between UPFs intake and other indices of lipid profile. Also, we found significant associations between UPFs intake and dietary nutrient profiles. In conclusion, UPFs consumption could worsen the nutritional profile of the diet and lead to negative changes in some indices of the lipid profile.

## Introduction

Dyslipidemia is defined as any lipid abnormality including elevated total cholesterol (TC), low-density lipoprotein cholesterol (LDL-C) and triglycerides (TG), and declined high-density lipoprotein cholesterol (HDL-C)^1^. Prevalence of hypertriglyceridemia, hypercholesterolemia, high non-HDL, and low HDL in Iranian adults are 28%, 26.7%, 39.5,% and 69.2% respectively^2^.

Abnormalities of lipid profile are associated with many clinical outcomes such as type 2 diabetes and cardiovascular diseases (CVDs). It is also the main cause of more than half of the cases of congenital heart disease and more than four million deaths annually^3^. There is a complex interaction between genetic factors and several environmental factors such as smoking, sedentary lifestyle, and socioeconomic situation as determinants of lipid profile^4^. Particularly taking foods rich in calories, carbohydrates, sodium, cholesterol, trans and saturated fatty acids are related to the high concentration of TC and LDL-C. On the other hand, consumption of polyunsaturated fatty acids, vegetables, dietary fibers, milk, and dairy products could decrease the risk of dyslipidemia^5^.

Nova classification system by considering physical, biological, chemical properties of the foods, also the additives for food manufacturing, classifies foods into different groups, including unprocessed and minimally processed food, processed culinary ingredients, processed foods and ultra-processed foods. UPFs are intrinsically fatty, sugary or salty, high calorie, and poor in protein, dietary fiber, micronutrients, and other bioactive compounds, and usually contain no or small amount of whole foods^6,7^. UPFs contain soft drinks; sweets like chocolate, candies, ice cream, biscuits and cakes; packaged leaves of bread; nugget and sticks, margarine, pastries; pre-prepared food dishes, and many other products^8^.

Evidence also showed that high consumption of UPFs is potentially related to the high rate of obesity and related comorbidities^9^. Assessment of UPFs’ effects on lipid profiles and upcoming diseases is an ongoing debate. Most studies reported a positive association of UPFs intake with lipid abnormalities, altered blood lipid factors varies in the studies. While, some reported association between UPFs intake with elevated TG and lower HDL-C^10–12^, others reported positive association of UPFs consumption with high LDL and total cholesterol^13,14^. As UPFs production and consumption have increased extremely during the last decades, comprehending their potential effects on human health has become a major concern in health care systems. The purpose of this cross-sectional study was to examine the association between UPFs intake and lipid profile in Iranian adults. In addition, as secondary outcomes, we examined the association of UPFs consumption with the intake of food subgroups, macronutrients, and some of the micronutrients. To the best of our knowledge, no previous study has investigated the association between UPFs intake and lipid profile in Iranian adults.

## Methods

### Study population

**Figure 1:**
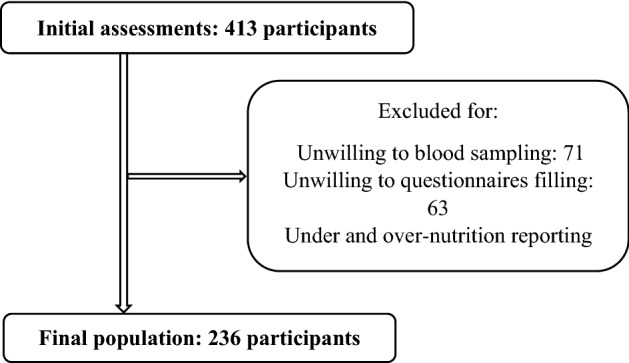
Study flow diagram.

This cross-sectional study was performed on 236 individuals with the age range of 20–50 years who were selected by cluster random sampling in health care centers of Shiraz-Iran (Fig. 1). For this purpose, Shiraz was divided in four clusters and one health care center was randomly selected in each cluster. Study sample size was calculated by the following formula, in which α = 0.01, β = 0.10 and r = ± 0.25.

$${\text{C}}=0.5 \times \text{ln} \left[\frac{(1+r)}{(1-r)}\right]$$

$$\text{N}=3+{\left[\left({\text{Z}}_{1-\frac{{\upalpha }}{2}}+{\text{Z}}_{1-\upbeta }\right)\div {\text{C}}\right]}^{2}$$

Prerequisites for participating in this study were no history of chronic diseases and no adherence to any special diet (The detailed of this study have been previously published^15,16^). Written consent form was signed by all participants. The protocol of this study was authorized by Shiraz University of Medical Sciences (IR.SUMS.REC.1394.S146).

### Dietary assessment

Food intakes of the participants were evaluated by a 168-item food frequency questionnaire (FFQ) which was previously validated in Iranian populations^17^. The FFQ was filled out based on family food intakes and data was changed to gram. For computing energy, and nutrients intakes we used NUTRITIONIST IV (version 7.0; N-Squared Computing, Salem, OR, USA).

To estimate the ultra-processed foods intake, classification of NOVA food group was used^8,18^. Accordingly, total daily consumption of some foods and beverages items were considered as ultra-processed food (including: packaged breads, buns, confectionaries, pastries and sweets, ice cream, biscuits, cakes, soft drinks, industrial fruit drinks, sweetened milk-based beverages, margarine, sauces, dressings, processed meats, fries, and salty snacks^19^). To define the contribution of every food group to overall consumption of ultra-processed foods, the mean daily intake of every 9 subgroups of ultra-processed foods (dairy products, non-dairy beverages, margarine and sauces, cakes and cookies, chips and snacks, breads, fast foods and meats, sweets and others) was divided by daily consumption of ultra-processed foods, then multiplied by 100.

### Biochemical assessments

Serum lipids including TC, TG, HDL-C and LDL-C were measured in blood samples (5 cm^3^) taken from the participants commercially accessible enzyme kits (Pars Azmoon, Tehran, Iran).

### Socio-demographic and anthropometric assessments

Sex, age, smoking habit and alcohol intake were evaluated by using a questionnaire. Also we used International Physical Activity Questionnaire (IPAQ)^20^ to assess physical activity level of the participants. Anthropometric indices [weight (kg), waist circumference (cm) and height (cm)] were measured. Then, according to weight and height, BMI was calculated.

### Statistical analysis

All analysis was done using SPSS for windows (version 20.0, SPSS Inc. Chicago IL, USA). The level of significance was P-value < 0.05. Normal distribution of the variables was checked by Kolmogorov–Smirnov test. The relationship between quantitative variables and qualitative variables was evaluated by analysis of variance test and Chi-square test respectively. Crude and adjusted models of logistic regression were used to evaluate the relation between UPFs score with anthropometric index and lipid profile. In adjusted models, the effects of age, energy intake, physical activity, BMI, sex and smoking history were controlled. We dichotomized lipid profile and anthropometric indices then LDL-C more than 130 mg/dL, HDL-C less than 40 mg/dL for male and 50 mg/dL for female, TC more than 200 mg/dL, TG more than 150 mg/dL, non-HDL more than 130 and WC more than 88 cm for female and 102 for male were considered as abnormal^16,21–24^.

### Ethical approval

The present study was approved by The Research Ethics Committee of Shiraz University of Medical Sciences, Shiraz, Iran (IR.SUMS.REC.1394.S146).

### Use of human participants

All experiments were performed in accordance with relevant guidelines and regulations.

### Informed consent

All participants singed informed consent form.

## Results

As shown in Table 1, mean age and BMI of study population were 45.97 years and 28.28, respectively kg/m^2^. Also, 58.90% of the study participants were female. According to Table 2 there was no significant difference between age, weight, height, BMI, waist circumference, WHR, TC, LDL, HDL, Non-HDL, physical activity, sex, smoking habits, alcohol intake history and education of the participants in the first and last tertile of UPFs, intake, but the difference was significant for mean of serum TG (P = 0.015).

**Table 1:** Baseline characteristics of the study participants.

Variables	N = 236
Age (year)	45.97 ± 11.74
Sex, female (%)	139 (58.90)
Education level, lower than high School (%)	65 (27.54)
Smoking history, yes (%)	28 (11.90)
BMI (kg/m^2^)	28.28 ± 4.69
Waist circumference (cm)	94.21 ± 11.35
Hip circumference (cm)	101.47 ± 9.64
WHR	0.90 ± 0.07
TG (mg/dL)	121.40 ± 64.44
TC (mg/dL)	179.82 ± 43.10
LDL-c (mg/dL)	109.33 ± 33.74
HDL-c (mg/dL)	38.01 ± 11.08
Non HDL-c	141.81 ± 40.53
Energy (kcal/day)	2772.84 ± 1054.18
Protein (g/day)	90.00 ± 37.12
Carbohydrate (g/day)	432.03 ± 168.19
Total fat (g/day)	82.70 ± 38.95
UPFs (kcal/day)	259.40 ± 289.15

*BMI* body mass index, *WHR* waist to hip ratio, *TG* triglyceride, *TC* total cholesterol, *LDL* low density lipoprotein, *HDL* high density lipoprotein, *UPFs* ultra-processed foods.

Values are mean ± SD for continuous and number (percentage) for categorical variables.

**Table 2:** Baseline characteristics according to tertile of UPFs.

Variables	UPFs			
	T_1_ (n = 95)	T_2_ (n = 79)	T_3_ (n = 62)	P-value
Age (year)	46.47 ± 10.92	46.68 ± 12.91	44.30 ± 11.41	0.427
Weight (kg)	73.37 ± 14.84	75.12 ± 13.98	76.34 ± 13.86	0.433
Height (cm)	162.23.18 ± 10.08	162.29 ± 9.00	164.53 ± 9.60	0.114
BMI (kg/m^2^)	28.14 ± 4.85	28.51 ± 4.78	28.18 ± 4.36	0.865
Waist circumference (cm)	93.81 ± 11.68	94.11 ± 11.11	94.95 ± 11.29	0.830
WHR	0.90 ± 0.08	0.89 ± 0.07	0.91 ± 0.07	0.333
TG (mg/dL)	109.92 ± 52.55	120.39 ± 66.75	140.27 ± 74.00	**0.015**
TC (mg/dL)	179.12 ± 47.21	178.60 ± 40.90	182.45 ± 39.65	0.854
LDL-C (mg/dL)	111.42 ± 36.74	106.55 ± 32.82	109.69 ± 30.19	0.638
HDL-C (mg/dL)	38.06 ± 11.59	39.24 ± 11.11	36.37 ± 10.18	0.313
Non-HDL	141.06 ± 44.42	139.36 ± 38.13	146.08 ± 37.43	0.606
Physical activity (Met.h/day)	21.56 ± 4.75	23.60 ± 4.20	17.23 ± 4.46	0.646
Sex (%)				0.735
Male	35.1	29.9	35.1	
Female	35.3	33.1	31.7	
Smoking history (%)				0.956
Yes	32.1	32.1	35.7	
No	35.6	31.7	32.7	
Education				0.093
Less than high school (%)	39.4	34.3	26.3	
High school and higher (%)	31.6	30.1	38.3	

*T* tertile, *UPF* ultra-processed food, *BMI* body mass index, *WHR* waist to hip ratio, *TG* triglyceride, *TC* total cholesterol, *LDL* low density lipoprotein, *HDL* high density lipoprotein.

Values are mean ± (SD) for continuous and percentage for categorical variables.

Using one-way ANOVA for continuous and Chi-square test for categorical variables.

Significant values are in bold.

Compared to the first tertile, individuals in the last tertile of UPFs intake had higher consumption of energy (P = 0.008), carbohydrate (P = 0.028), fat (P = 0.002), cholesterol (P = 0.023), SFA (P = 0.004), MUFA (P = 0.001), PUFA (P = 0.005), vitamin B_12_ (P = 0.013) and vitamin C (P = 0.027). But, we observed no significant association between intake of vitamin B_6_, B_9_, C, calcium, magnesium, zinc and selenium with UPFs intake (Table 3).

**Table 3:** The study participants’ nutrients intakes among the tertiles of UPFs.

Variable	UPFs			
	T_1_ (n = 95)	T_2_ (n = 79)	T_3_ (n = 62)	P-value
Energy (kcal/day)	2756.65 ± 985.99	2542.11 ± 876.50	3091.63 ± 1275.98	**0.008**
Carbohydrate (g/day)	434.77 ± 164.46	396.90 ± 144.51	472.59 ± 193.16	**0.028**
Protein (g/day)	89.29 ± 33.10	83.85 ± 98.94	98.95 ± 59.02	0.055
Fat (g/day)	80.12 ± 35.40	74.57 ± 30.00	97.02 ± 49.59	**0.002**
Cholesterol (mg/day)	212.82 ± 118.46	203.34 ± 81.51	258.33 ± 167.20	**0.023**
SFA (mg/day)	23.28 ± 11.86	19.87 ± 7.28	26.10 ± 13.72	**0.004**
MUFA (mg/day)	25.76 ± 12.32	23.50 ± 9.57	31.54 ± 17.23	**0.001**
PUFA (mg/day)	18.63 ± 9.28	17.63 ± 8.20	22.98 ± 13.02	**0.005**
Vitamin B_6_ (mg/day)	2.22 ± 0.86	2.01 ± 0.74	2.26 ± 0.97	0.159
Vitamin B_9_ (µg/day)	717.53 ± 253.30	692.82 ± 223.75	784.71 ± 296.37	0.099
Vitamin B_12_ (µg/day)	3.53 ± 1.84	3.34 ± 1.59	4.35 ± 2.93	**0.013**
Vitamin C (mg/day)	213.13 ± 159.96	168.96 ± 87.76	222.39 ± 122.48	**0.027**
Vitamin E (mg/day)	14.81 ± 7.72	13.42 ± 4.47	16.15 ± 7.29	0.055
Calcium (mg/day)	1170.07 ± 478.76	1068.75 ± 423.82	1191.43 ± 437.45	0.203
Magnesium (mg/day)	481.70 ± 224.14	441.32 ± 207.59	493.62 ± 224.30	0.312
Zinc (mg/day)	13.29 ± 5.83	12.52 ± 5.64	14.13 ± 7.18	0.306
Selenium (mg/day)	130.70 ± 74.55	119.30 ± 61.64	133.08 ± 71.68	0.431

*T* tertile, *UPFs* ultra-processed foods.

Values are mean ± (SD).

Using one-way ANOVA.

Significant values are in bold.

As presented in Table 4**,** participants in the last tertile of UPFs intake had significantly higher intakes of processed and red meat (P = 0.007) and breads (P = 0.022), also lower intake of margarine and sauces (P < 0.001). There were no differences in non-dairy beverages, cookies and cakes, dairy products, potato chips and salty snacks, sweets and other food items intakes among the UPFs tertiles.

**Table 4:** Daily intakes of UPFs subgroups across the tertiles of UPFs.

Variables	UPFs			
	T_1_ (n = 95)	T_2_ (n = 79)	T_3_ (n = 62)	P-value
Non-dairy beverage (%)	11.94 ± 1.69	9.75 ± 1.58	11.61 ± 1.53	0.593
Cookies and cakes (%)	19.91 ± 2.16	19.84 ± 2.29	23.48 ± 2.21	0.452
Dairy products (%)	11.34 ± 1.66	10.27 ± 1.18	8.68 ± 1.09	0.376
Potato chips and salty snacks (%)	7.52 ± 1.06	5.42 ± 1.27	6.14 ± 1.17	0.438
Processed meat and fast food (%)	7.01 ± 1.06	10.40 ± 1.49	13.42 ± 1.77	**0.007**
Margarine and sauces (%)	16.55 ± 1.51	15.70 ± 1.37	7.99 ± 0.70	˂ **0.001**
Sweets (%)	5.42 ± 0.82	5.72 ± 1.31	3.10 ± 0.46	0.091
Bread (%)	5.99 ± 0.96	5.17 ± 0.86	10.57 ± 2.20	**0.022**
Others (%)	14.33 ± 1.93	17.29 ± 2.12	15.01 ± 1.97	0.556

*T* tertile, *UPFs* ultra-processed food.

Values are mean ± (SD).

Using one-way ANOVA.

Significant values are in bold.

We found that, higher UPFs intake was associated with increased OR of serum TG and HDL abnormality in both crude (OR 3.41; 95% CI 1.58, 7.34; P-trend = 0.001 and OR 2.99; 95% CI 1.31, 6.82; P-trend = 0.010) and adjusted model (OR 3.69; 95% CI 1.67, 8.16; P-trend = 0.001 and OR 3.38 95% CI 1.42, 8.07; P-trend = 0.009) (Table 5).

**Table 5:** Crude and multivariable-adjusted odds ratios and 95% CIs for lipid profile across UPFs tertile.

Variables	UPFs					
	T_1_	T_2_	P	T_3_	P	P_trend_
TG (mg/day)						
Crude model	Ref.	1.52 (1.02, 5.30)	**0.015**	3.41 (1.58, 7.34)	**0.002**	**0.001**
Adjusted model	Ref.	2.54 (1.19, 5.43)	**0.016**	3.69 (1.67, 8.16)	**0.001**	**0.001**
TC (mg/day)						
Crude model	Ref.	0.91 (0.44, 1.86)	0.796	1.58 (0.77, 3.22)	0.210	0.243
Adjusted model	Ref.	1.03 (0.49, 2.14)	0.937	1.60 (0.77, 3.35)	0.206	0.230
LDL (mg/day)						
Crude model	Ref.	1.04 (0.52, 2.12)	0.890	1.58 (0.77, 3.22)	0.210	0.228
Adjusted model	Ref.	1.08 (0.53, 2.20)	0.827	1.57 (0.76, 3.26)	0.221	0.239
HDL (mg/day)						
Crude model	Ref.	1.36 (0.68, 2.52)	0.408	2.99 (1.31, 6.82)	**0.009**	**0.010**
Adjusted model	Ref.	1.11 (0.57, 2.18)	0.748	3.38 (1.42, 8.07)	˂ **0.001**	**0.009**
Non-HDL						
Crude model	Ref.	0.97 (0.53, 1.77)	0.924	1.10 (0.57, 2.12)	0.770	0.795
Adjusted model	Ref.	0.94 (0.50, 1.75)	0.854	1.07 (0.54, 2.11)	0.830	0.866
WC (cm)						
Crude model	Ref.	0.77 (0.42, 1.41)	0.400	0.86 (0.45, 1.65)	0.665	0.604
Adjusted model	Ref.	0.68 (0.23, 1.94)	0.473	1.50 (0.48, 4.73)	0.481	0.611
WHR						
Crude model	Ref.	0.70 (0.36, 1.34)	0.300	1.05 (0.51, 2.15)	0.893	0.986
Adjusted model	Ref.	0.67 (0.31, 1.44)	0.309	1.08 (0.48, 2.46)	0.837	0.951

*T* tertile, *UPFs* ultra-processed foods, *TG* triglyceride, *TC* total cholesterol, *LDL* low density lipoprotein, *HDL* high density lipoprotein, *WC* waist circumference, *WHR* waist to hip ratio.

Adjusted for age, energy intake, BMI, smoking and sex.

These values are odds ratio (95% CIs).

Obtained using logistic regression.

Significant values are in bold.

## Discussion

We found that higher intake of UPFs was associated with elevated lipid profile abnormality including TG and HDL. In terms of other blood lipids such as TC, LDL and non-HDL, while they were not significantly associate with UPFs intake, all were higher in last tertile of UPFs intake.

In agreement with our study, Lima et al. reported that higher UPFs consumption was associated with higher TG and lower HDL-C levels^25^. Furthermore, a large cohort of older adults in Spain found the same results^10^, and a systematic review and meta-analysis resulted in a negative association between UPFs consumption and HDL-cholesterol levels^12^. On the other hand, a cross-sectional study on Ecuadorian adolescents showed that dietary patterns consisted of processed foods were associated with an increased level of LDL and cholesterol^13^. Also, a longitudinal study in Brazil on preschool children found that intake of UPFs was a predictor of higher total cholesterol and LDL cholesterol but not HDL and TG probably because effects of food habits on serum LDL and total cholesterol in children are stronger than its effects on other dyslipidemia markers^14^.

We found a strong association between the dietary contribution of UPFs and the dietary content of energy, carbohydrates, fat, cholesterol, and SFAs. Moreover, intake of MUFAs and PUFAs increased parallel to UPFs consumption, probably due to high fat content of the UPFs.

With regard to anthropometric indices, although WC and WHR were not significantly associated with UPFs intake, participants in the last tertile of UPFs had higher mean of WC and WHR. Findings of some cohort studies, indicated that higher UPFs intake was associated with greater adiposity accumulation, higher BMI, weight gain and incidence of obesity^26–28^. Besides, another cohort study on overweight and obese participants aged 55–75 years reported that higher UPFs intake was associated with higher age-related increase in visceral and overall adiposity^29^. Another similar study also showed a positive association between UPFs intake and the incidence of abdominal obesity in adults^30^. A systematic review and meta-analysis and a multi-national cohort study also reported positive associations between higher consumption of UPFs and general and abdominal obesity^12,31^. We assume that our opposite findings is due to the high mean of our participants’ weight, WHR, and WC.

UPFs are often calorie-dense, contain large amounts of fats, saturated fats, trans fats, sodium, and simple sugars with high glycemic index and contain no or small amount of fibers, vitamins, minerals, or other bioactive compounds which naturally exist in fresh foods, so they are nutritionally unbalanced^32^.

Several mechanisms have been suggested to explain the adverse effects of UFPs on lipid profile and health. First of all, due to their intrinsic palatability, overconsumption of UPFs may result in physiological disruption of hunger and satiety patterns^33–36^. Besides, energy-dense foods usually contain high amounts of free sugar and trans fats which enhance lipogenesis, and decrease fatty acids oxidation which lead to their aggregation in tissues and blood circulation and elevated LDL level^37^. Furthermore, partially hydrogenated vegetable oils content of UPFs contain *trans* fatty acids which have adverse impacts on lipid profile. In addition, scientific evidence support the hypothesis that the interacting effects of substances produced through the high-heat processing of oils, determine their health effects^38^. Heat processing also causes degradation of food substances and formation of furans in UPFs^39^. Higher intake of UPFs increase exposure to phthalates which is used in the packing process and could transfer to food^40^. The accumulation of phthalates, bisphenols, furans, and their metabolites may ultimately lead to lower HDL-c and elevated TG levels through disruption of endocrine functions^41,42^. Evidence has shown that urinary concentration of phthalates and their metabolites was positively associated with TG and negatively associated with LDL levels^43^.

On the other hand, the impact of carbohydrates on lipid profile also depends on their sources and processing methods. While consumption of free sugar elevates serum TG, whole grains intake decreases TC, LDL and TG levels^44^. Consumption of minimally-processed whole grains such as oatmeal, instead of highly processed refined grains may improve lipid profile^45^.

Our study has some limitations as follows: First, because of the nature of the cross-sectional study, we were not able to assess causal correlations between UPFs intake and lipid profile. In the second place, the study was done in Shiraz city, so it should be generalized to other Iranian adults with caution. Ultimately, although we have removed the effects of some confounders in our analysis, there may be some others which have not been recognized in this study.

In conclusion, our results showed significant associations between ultra-processed foods intake and dietary nutrient profiles which resulted in dyslipidemia as a risk factor for chronic diseases. Findings of the present study highlight a necessity for more evidence, particularly longitudinal, to define the effect of UPFs on lipid profiles.

## Data Availability

Data available on request from the corresponding author.
